# Altered Functional Connectivity of Cerebello-Cortical Circuit in Multiple System Atrophy (Cerebellar-Type)

**DOI:** 10.3389/fnins.2018.00996

**Published:** 2019-01-04

**Authors:** Shan Ren, Hao Zhang, Weimin Zheng, Ming Liu, Fang Gao, Zhiqun Wang, Zhigang Chen

**Affiliations:** ^1^Department of Neurology, Dongfang Hospital, Beijing University of Chinese Medicine, Beijing, China; ^2^Department of Radiology, Dongfang Hospital, Beijing University of Chinese Medicine, Beijing, China; ^3^Department of Neurology, Xuanwu Hospital, Capital Medical University, Beijing, China

**Keywords:** resting state fMRI, functional connectivity, multiple system atrophy, amplitude of low-frequency fluctuation, network

## Abstract

Multiple system atrophy (MSA) is regarded as a progressive neurodegenerative disease mainly divided into MSA-p type with Parkinsonism and MSA-c type with cerebellar ataxia as the main symptom. However, its neural mechanism is still unclear. In this study, we only focus on the MSA-c type. The purpose of this study is to explore the functional connectivity changes of the cerebello-cortical circuit in MSA-c type by using resting state functional magnetic resonance imaging (rs-fMRI). Thirty-six subjects (18 MSA and 18 normal controls) participated in this study and the rs-fMRI data were collected by applying resting state amplitude of low-frequency fluctuations (ALFF), we found the significant decreased ALFF in the MSA patients relative to controls, which included left cerebellum 8 area, 9 area, 7b area and Cru1 as well as vermis 7. Then we select the brain region of cerebellum 8 area as seed to investigate whole brain functional connectivity alteration in the MSA patients. When comparing to controls, several regions showed decreased connectivity in the MSA patients including bilateral cerebellum anterior lobe, left cerebellum posterior lobe, left dentate, bilateral pons, inferior parietal lobule (IPL), lingual gyrus (LG), parahippocampus (PHG), and middle temporal gyrus (MTG). In addition, there were closely correlation between functional connectivities and clinical performances in the MSA patients. The current study confirmed that the disrupted functional connectivity of specific cerebello-cortical circuit in the MSA patients, which is responsible for the clinical performances.

## Introduction

Multiple system atrophy (MSA) is regarded as a progressive neurodegenerative disease characterized by dysautonomic nervous system, Parkinsonism with low dopamine response and cerebellar ataxia (Krismer and Wenning, 2017). Currently, it is mainly divided into MSA-p type with Parkinson’s disease as the main symptom, and MSA-c type with cerebellar ataxia as the main symptom (Gilman et al., 2008). The most important view of MSA pathogenesis is the appearance of the alpha-synuclein in cytoplasm of glial cells, leading to the degeneration of neuron myelin, the activation of microglial cells, and the induction of oxidative stress, which ultimately leads to thedeath of neuron (Brettschneider et al., 2017). From the perspective of functional integration, the hypothesis of “disconnection syndrome” indicated that the accumulation of alpha-synuclein may damage the specific functional connectivity networks including the striatal-thalamo-cortical (STC) and cerebello-thalamo-cortical (CTC), causing corresponding functional disorders of MSA (Yao et al., 2017; Rosskopf et al., 2018). However, the evidence of the network changes in MSA is scarce.

Recently, increasing attention has focused on exploring MSA-related intrinsic brain activity and connectivity changes during resting-state functional magnetic resonance imaging (rs-fMRI) (Fox and Greicius, 2010). For the intrinsic brain activity, by using the regional homogeneity (ReHo) approach, the researcher found that motor related cortices were functionally altered and played an important role in motor network dysfunction in MSA patients (You et al., 2011). By analysis of low-frequency fluctuations, another study explored the abnormality of spontaneous brain activity in MSA-p type, which was involved in the STC network, default mode network (DMN), visual related cortices and cerebellum (Wang et al., 2017). By applying the functional connectivity of cerebellar dentate nucleus, a recent study demonstrated a crucial role for the CTC network in addition to STC network in MSA-p patients, which indicated the degeneration of cerebello-cortical network in the development of the disease (Yao et al., 2017). By using the defined seed-based correlation analysis, another recent study revealed increased pontocerebellar functional connectivity and decreased DMN connectivity in MSA patients (Rosskopf et al., 2018). However, no investigations explored the distinct network pattern on MSA by combining regional activity and functional connectivity analysis. And furthermore, most previous studies focused on the MSA-p type, few studies explored the cerebellar network changes in the MSA-c type. Here, we plan to explore the network changes of MSA-c type by using both regional activity and functional connectivity analysis.

In this study, in order to reflect regional spontaneous brain activity, we first used the amplitude of low-frequency fluctuations (ALFF) method to examine the whole brain activity changes of MSA-c type patients during resting state. The ALFF values were acquired by calculating the square root of the power spectrum of the rs-fMRI signals in a low-frequency range (usually 0.01–0.08 Hz) (Zang et al., 2007). Second, we selected the region presenting most significantly different ALFF as seed and investigated the functional connectivity of the region using a seed-based approach. ALFF is used as functional segregation analysis to reveal the regional intrinsic brain activity, while seed-based connectivity analysis gives measures of functional integration. Based on the two techniques, we sought to investigate whether regional activity and connectivity was impaired in MSA-c type patients compared to healthy controls. The cerebellar cortex send the efferent fibers to dentate nuclei, and then the neural fibers form the main body of the superior cerebellar peduncles, which project into the contralateral thalamus, and then pass through the thalamus to the motor related cerebral cortex, thus completing the cerebello-cortical circuits (Middleton and Strick, 2000), which might be destroyed by the pathological changes of MSA-c type. Base on the previous study, we hypothesized that the most significant ALFF changes of the MSA-c type patients might be located in the cerebellum. In addition, the functional connectivity between the selective region and the whole brain cortical or subcortical regions might constitute the cerebello-cortical circuits, which may be disrupted in the MSA c-type patients.

## Materials and Methods

### Participants

All subjects were recruited from Dongfang Hospital of Beijing University of Chinese Medicine. Data of 20 MSA-c type patients and 20 normal subjects were collected in this study. The diagnosis of MSA-c type fulfilled the criteria for probable MSA-c type based on the American Academy of Neurology and American Autonomic Society (Gilman et al., 2008). Four subjects (two MSA-c type patients and two controls) were excluded due to excessive head motion during the fMRI preprocessing, yielding a total of 18 MSA-c type patients and 18 controls for the final analysis. All subjects were assessed by complete physical and neuropsychological examinations including Mini-Mental State Examination (MMSE) and Montreal Cognitive Assessment (MoCA). All the MSA patients were evaluated by the Unified Multiple System Atrophy Rating Scale (UMSARS), which was conducted to assess the severity of the illness. The clinical examinations were performed on the day before fMRI scanning.

The inclusion criteria for controls were as follows: (1) there was no neurological or psychiatric disorders including stroke, depression or epilepsy; (2) there was no neurological deficiencies including visual or hearing loss; (3) there was no abnormal findings of brain magnetic resonance imaging (MRI), such as infarction or focal lesions.

The subjects who suffered from hemorrhage, infarction, tumors, trauma, or severe white matter hyperintensity were excluded from the study. Clinical and demographic information for the subjects was shown in Table 1. This study was carried out in accordance with the recommendations of the Medical Research Ethical Committee of Dongfang Hospital of Beijing University of Chinese Medicine with written informed consent from all subjects. All subjects gave written informed consent in accordance with the Declaration of Helsinki. The protocol was approved by the Medical Research Ethical Committee of Dongfang Hospital of Beijing University of Chinese Medicine.

**Table 1:** Demographic and clinical characteristics of the participants.

Characteristics	MSA-c type (*n* = 18)	Control (*n* = 18)	*p*-Value
Age, years	57.56 ± 1.34	57.61 ± 1.19	0.376
Gender, male/female	8/10	7/11	0.735
Education, years	13.78 ± 0.46	13.72 ± 0.49	0.183
Disease duration, years	4.28 ± 0.19	NA	
MMSE	27.00 ± 1.68	27.56 ± 1.79	0.344
MoCA	27.83 ± 1.20	28.28 ± 1.18	0.270
UMSARS-I, total	18.39 ± 1.31	NA	
UMSARS-II, total	17.61 ± 1.41	NA	
Over disability grade	2.50 ± 0.22	NA	


### Data Acquisition

MRI data acquisition was performed on a GE 3.0T Discovery 750 scanner. Foam padding and headphones were used to control head motion and scanner noise. Functional images were collected with the following parameters: repetition time (TR)/echo time (TE)/flip angle (FA) = 2000 ms/30 ms/90°, field of view = 24 cm × 24 cm, resolution = 64 × 64 matrix, slices = 36, thickness = 3 mm, gap = 1 mm, voxel size = 3.75 mm × 3.75 mm × 3 mm, and bandwidth = 2,232 Hz/pixel. During the scan, subjects were required to hold still, keep their eyes closed and think of nothing in particular. A simple questionnaire after the scan was performed to confirm that none of subjects had fallen asleep. For registration purposes, high-resolution anatomical images were collected using a 3D brain volume (BRAVO) T1-weighted sequence with the following parameters: TR/TE/inversion time (TI)/FA = 8150 ms/3.17 ms/450 ms/12°, resolution = 256 × 256 matrix, slices = 188, thickness = 1 mm, voxel size = 1 mm × 1 mm × 1 mm.

### Data Preprocessing

Functional MRI data preprocessing were performed using statistical parametric mapping (SPM) 12 software^1^ and data processing assistant for resting-state fMRI (DPARSF)^2^ (Yan and Zang, 2010) toolkits, which included the first 10 volumes deletion, slice timing correction and head motion correction. In order to spatially normalize the fMRI data, the functional data is recorded to its corresponding anatomical image using a T1-weighted image, and the resulting aligned T1 data set is converted to Montreal Neurological Institute (MNI) space (Ashburner and Friston, 2005). Then to improve the registration of fMRI data, a custom T1 template was constructed by averaging standardized anatomical images of all subjects. Last, the normalized functional images were created by applying a template created by T1 images transformation. The functions of the images were resampled to 3 mm isotropic voxel and spatial smoothed with a 4 mm full width at half maximum (FWHM) Gaussian kernel. To reduce the effects of low frequency drift and high frequency physiological noise, linear detrending and time band pass filtering (0.01–0.08 Hz) were performed. Finally, several disturbing variables, including parameters of head motion, white matter, cerebrospinal fluid (CSF) signal, and global average signal were regressed by multiple linear regression analysis. During image preprocessing, four subjects were excluded due to excessive head motion (translation > 2 mm, rotation > 2°).

### ALFF Analysis

ALFF analysis was performed using the REST software^3^ with a voxel-based approach. ALFF analysis was performed using the DPARSF software. After preprocessing, time band-pass filtering (0.01–0.08 Hz) of the fMRI data reduced the effects of a low-frequency drift and high frequency physiological noise, such as respiratory and cardiac rhythms. The time series of each voxel was converted to the frequency domain using fast Fourier transform (FFT) (parameters: taper percent = 0, FFT length = shortest) and a power spectrum was obtained. The square root was computed at each frequency of the power spectrum because the power of a given frequency was proportional to the square of the amplitude of the frequency component, and then an average square root of 0.01–0.08 Hz per voxel was obtained. The average square root was regarded as ALFF, which was assumed to reflect the absolute intensity of spontaneous activity of the brain, and reflected the level of spontaneous activity of each voxel at rest in terms of energy.

### Functional Connectivity Analysis

To investigate change in network level function in MSA-c type patients, we conducted a seed-based inter-regional correlation analysis study. According to the ALFF result, there might be several regions identified as significant abnormalities in patients with MSA-c type. We selected the region presenting most significant difference between the two groups as a cluster mask, and defined it as region of interest for functional connectivity analysis. Correlation analysis between the time series of seed and the time series of the entire brain in a voxel-wise way was performed. The value of z is obtained using Fisher’s r-to-z transformation to improve the Gaussianity of its distribution.

### Statistical Analysis

The statistics analysis was performed using SPM12 software. First, for the resting state between-group ALFF comparison (between the MSA-c type patients and controls), a two-sample *t*-test was performed on the individual normalized ALFF maps. Multiple comparison corrections used the Family Mode Error (FWE) method with a threshold *P* < 0.05.

Second, to assess the differences of the whole-brain connectivity of selected region, two-sample *t*-test was used to compare the between-group differences (*P* < 0.05, FWE correction) in MSA-c type patients and controls, with gender and age as covariates.

Third, to investigate the relationship between the strength of the connection and the UMSARS score, the Pearson correlation test was performed to determine the correlation between the functional connectivity changes of selected regions and the clinical variables as measured by UMSARS. *P*-values were compared for multiple comparisons using the Bonferroni method correction.

## Results

### Demographic and Neuropsychological Tests

Demographic and clinical characteristics are described in Table 1. No significant differences of gender, age, education, MMSE and MoCA scores were found between the MSA-c type and control groups. However, the MSA-c type group exhibited increased UMSARS scores which refer to the severity of the disease.

### ALFF Changes Between the MSA-c Type and Controls in the Resting State

**FIGURE 1:**
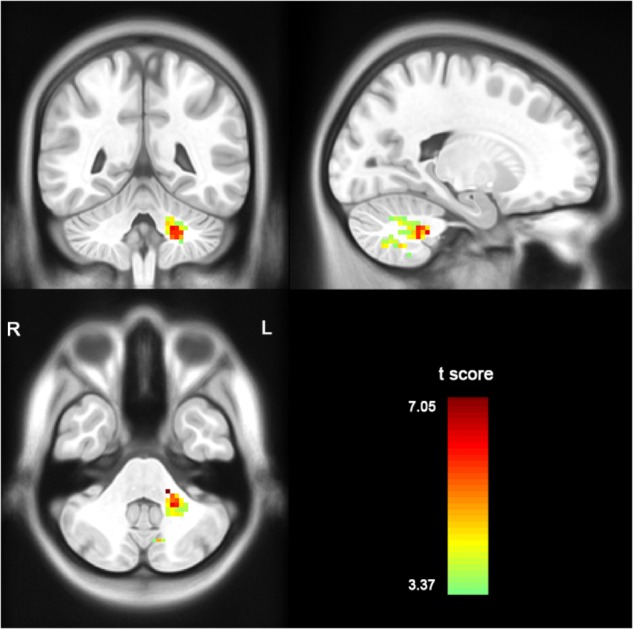
Between-group differences of ALFF value in MSA-c type and controls in the resting state, based on a two-sample *t*-test. Multiple comparison corrections used the Family Mode Error (FWE) method with a threshold *P* < 0.05. The color scale represents *t*-values. Cold color represents decreased ALFF in MSA-c type patients compared to controls. MSA, multiple system atrophy; ALFF, amplitude of low-frequency fluctuations.

Compared with the healthy controls, the patients with MSA-c type presented significantly decreased ALFF in left cerebellum 8 area, 9 area, 7b area and Cru1 as well as vermis 7. The peak voxels within those significantly different clusters were shown in Figure 1 and Table 2.

**Table 2:** Regions of significant different ALFF between MSA-c type and controls.

Brain regions	Cluster voxels	MNI coordinates (mm)			*t* score
		*x*	*y*	*z*	
L. cerebellum 8 L. cerebellum 9	60	-24	-50	-42	6.04692
	12	-19	-43	-41	6.04518
L. cerebellum 7b	9	-9	-72	39	4.81744
L. cerebellum Cru1	8	-12	-73	30	3.38457
Vermis 7	6	-3	-72	-30	4.82177


### Functional Connectivity Between MSA-c Type Group and Controls

**FIGURE 2:**
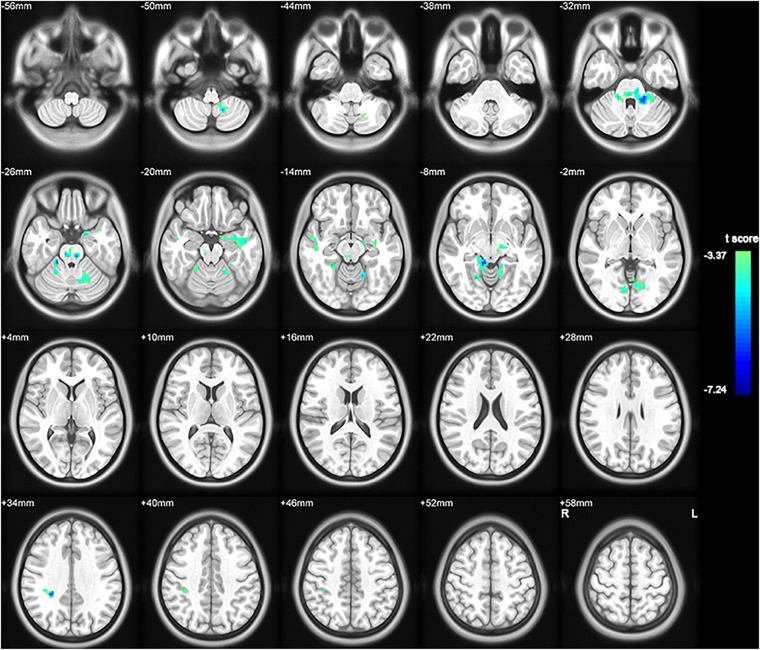
Between-group differences of functional connectivity in MSA-c type and controls in the resting state (*P* < 0.05, FWE correction). Region which was significantly changed in ALFF was selected as seed to perform whole brain connectivity. Two-sample *t*-test was used with gender and age as covariates. The color scale represents *t*-values. Cold color represents decreased functional connectivity in MSA-c type patients compared to controls. MSA, multiple system atrophy; ALFF, amplitude of low-frequency fluctuations.

**FIGURE 3:**
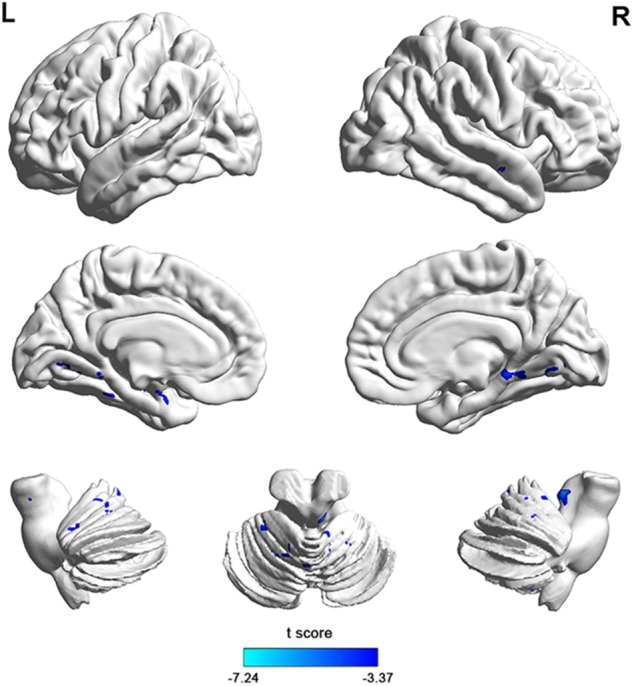
The 3D map showed between-group differences of functional connectivity in MSA-c type and controls in the resting state (*P* < 0.05, FWE correction). Two-sample *t*-test was used with gender and age as covariates. The color scale represents *t*-values. Cold color in the 3D map represents decreased functional connectivity in MSA-c type patients compared to controls. MSA, multiple system atrophy.

To investigate functional connectivity alterations in the MSA-c type patients, seed based interregional correlation was analyzed. We selected the region of left cerebellum 8, which were significantly changed in ALFF as seeds. Figures 2, 3 and Table 3 showed that the MSA-c type patients presented decreased connectivity between the left cerebellum 8 and several regions, including bilateral cerebellum anterior lobe, left cerebellum posterior lobe, left dentate, bilateral Pons, inferior parietal lobule (IPL), lingual gyrus (LG), parahippocampus (PHG), and middle temporal gyrus (MTG).

**Table 3:** Regions of significant different connectivity between MSA-c type and controls.

Brain regions	MNI coordinate			Cluster size	*t* score
	*x*	*y*	*z*		
R. cerebellum anterior lobe	9	-36	-6	92	-7.2377
L. cerebellum anterior lobe	9	-36	-6	105	-7.2377
L. and R. pons	9	-36	-6	81	-7.2377
L. dentate	9	-36	-6	26	-7.2377
R. inferior parietal lobule	30	-39	33	33	-5.7002
L. parahippocampus	-42	-6	-24	108	-5.4155
L. lingual gyrus	-18	-54	-18	10	-5.1332
L. cerebellum anterior lobe	-18	-54	-18	23	-5.1332
L. cerebellum posterior lobe	-15	-57	-51	35	-5.0224
R. lingual gyrus	21	-60	-9	52	-4.8816
R. cerebellum anterior lobe	-18	-54	-18	11	-4.8816
R. middle temporal gyrus	51	-3	-18	33	-4.2308


### Correlation Between Functional Activity and Clinical Performances in the MSA-c Type Group

**FIGURE 4:**
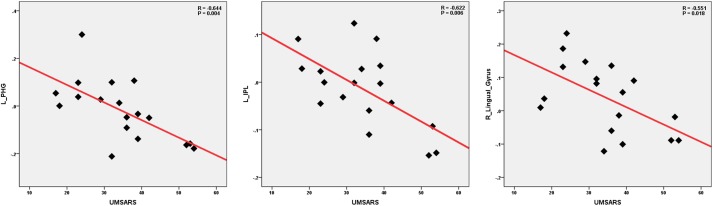
Correlations of clinical variables and cerebellum functional connectivity in MSA-c type patients. *P*-values were compared for multiple comparisons using the Bonferroni method correction. The UMSARS scores showed negative correlation with cerebellar connectivity of the several regions including the left PHG, IPL and right LG. MSA, multiple system atrophy; UMSARS, the Unified Multiple System Atrophy Rating Scale; PHG, parahippocampus; IPL, inferior parietal lobule, LG, lingual gyrus.

We performed the Pearson correlation to explore the strength of the connection and the clinical performances. For the strength of connectivity, we performed correlation analysis between the time series of seed and the time series of the other brain regions in a voxel-wise way. We extracted the value of z of each subject by using Fisher’s r-to-z transformation. Correlation coefficient of *z*-value reflect the functional connectivity strength. In the MSA-c type group, we found negative correlations between the UMSARS scores and connectivity of the left cerebellum 8 and the left PHG, IPL as well as right LG. besides UMSARS, we didn’t find the correlation between the connectivity and other clinical parameters. Figure 4 showed the relationship of clinical variables and cerebellum functional connectivity in MSA-c type patients.

## Discussion

By applying ALFF analysis to the resting state fMRI data acquired from MSA-c type and controls, we observed significant decreased ALFF values in several cerebellum regions in MSA-c type patients. By using the selected cerebellum 8 as seed, we showed distinctive pattern of functional connectivity the in MSA-c type, which exhibited functional disconnection between cerebellum 8 area with several regions including the cerebellum anterior and posterior lobe, dentate, Pons, IPL, LG and temporal lobe. Importantly, the disrupted patterns of cerebello-cortical circuits in some specific regions were significantly associated with UMSARS scores in the MSA-c type patients.

Decreased ALFF values were revealed in several cerebellum regions in MSA-c type patients, which predicted to the impaired intrinsic brain activity in the disease. Typical histopathological findings of MSA-c type have been observed predominantly in cerebellum (Brettschneider et al., 2017). By using voxel-based morphometry and diffusion tensor imaging method, recent study found that gray matter loss and white matter changes was limited in cerebellum and association tracts in the MSA-c type (Dash et al., 2018). Based on the association between structure and function, we speculated that the impaired intrinsic brain activity might be due to the structural atrophy of the cerebellum in the MSA-c type patients. Recently, ALFF has been used to explore the abnormalities in spontaneous neuronal activity in MSA patients. The previous study focused on MSA-p type and found that lower ALFF mainly in bilateral basal ganglion, as well as higher ALFF in right cerebellum and parieto-temporo-occipital cortex. The result is different from our result, which is due to the different type of the MSA patients. MSA-p type manifested predominantly as Parkinsonism and mainly targeted on the basil ganglion, while the MSA-c type presented cerebellar ataxia as the main symptom and emphasized on the cerebellum impairment.

We revealed the disrupted cerebello-cortical circuit in MSA-c type, which exhibited functional disconnection between cerebellum 8 with several regions including the cerebellum, dentate, Pons, IPL, LG and temporal lobe. As we know from cerebello-cortical circuit: The afferent fibers of cerebellum mainly come from the opposite cerebellopontine nucleus and the inferior olivary nucleus, respectively forming the cerebellopontine fiber bundle and the olivine cerebellum fiber bundle, passing through the middle and lower cerebellar peduncles to the new cerebellum; on the other hand, the cerebellar cortex send the efferent fibers to dentate nuclei, and then the neural fibers form the main body of the superior cerebellar peduncles, which project into the contralateral thalamus, and then pass through the thalamus to the motor related cerebral cortex. The network was responsible for the balance, planning and coordination of motor functions. From the result of our study, the cerebellum, dentate, Pons, IPL, LG and temporal lobe were involved in the cerebello-cortical circuit, which showed disrupted connectivity in the MSA-c type patients.

The dentate is the largest single structure connecting the cerebellum to the rest of the brain. By using dentate as seed region, several studies have found the disrupted connection between dentate and cortical or subcortical regions in the resting state in Parkinson’s disease (PD) (Liu et al., 2013; Ma et al., 2015) as well as MSA-p type (Yao et al., 2017), indicating impaired motor related symptoms. However, there has been no reported data about MSA-c type. Our study added new evidence of the cerebellum and dentate connectivity pattern on the MSA-c type. For the Pons, the afferent fibers of the cerebellum come mainly from the pontine nucleus. Based on the previous study, the connection between cerebellum and Pons played an important role in maintain body balance, regulate muscle tone and coordinate muscle movement. For the IPL function, several task-related fMRI studies have reported that the IPL played an important role in sensorimotor functions including object manipulation (Binkofski et al., 1999), tool use (Peeters et al., 2009), motor execution and imagery (Hanakawa et al., 2008). Previous resting state studies have consistently reported the existed functional connectivity between the cerebellum and parietal cortex (Liu et al., 2013; Macher et al., 2014). The functional disconnection between the cerebellum and the IPL may contribute to the impaired simple and complicated movement function of PD patients (Liu et al., 2013) as well as MSA-p type patients (Yao et al., 2017). We think there was the same condition for the MSA-c type patients.

Besides, we also found the disrupted connectivity between cerebellum and the temporal lobe including PHG and MTG in the MSA-c type. As we known, the PHG and MTG was the primary hub of the DMN during resting state, which functionally connected between the hippocampus and the other cortical cortices (Ward et al., 2014). The DMN is a functional-anatomic network involved in memory, mental imagery, self-reflection, and stream-of-consciousness processing and so on (Greicius et al., 2004; Yao et al., 2017). The majority of resting state fMRI studies in Alzheimer’s disease demonstrated the disruption of DMN, which contributed to the memory deficit (Zheng et al., 2017; Liu et al., 2018). However, in the previous study, changes in DMN connectivity have been demonstrated in several neurological disorders, including depression (Sheline et al., 2010), autism spectrum disorders (Assaf et al., 2010), schizophrenia (Rotarska-Jagiela et al., 2010), as well as in MSA-p type (Franciotti et al., 2015). From the view of clinical feature, these disorders do not mainly manifest as memory impairment. Therefore, the cortical DMN dysfunction probably not only limits to amnestic disorders, but also it may be a more general indicator of synaptic pathology (Buckner et al., 2008).

In addition, LG was also involved in the disrupted cerebellar connectivity in the MSA-c type patients. LG is located in the primary visual cortex and play a critical role in visual cognition. By using fMRI method, the higher activity of the region was revealed during the task of visuo-perceptual working memory (Sala-Llonch et al., 2015). We speculated the visual cognition deficits of the MSA patients might due to the disruption of the LG connectivity. Further study needs to be performed in the future.

The correlation analysis demonstrated that the connectivity of the left cerebellum and left PHG, IPL as well as right LG were negatively correlated with the behavior performance as measured by UMSARS. These findings suggested that functional activities of these regions contributed to the motor related function decline in the MSA-c type patients. These regions may play a crucial role in integrating information and mediating motor related functions.

There are still some issues to be noticed. First, it will be more helpful to set different MSA subtype to compare the resting state functional connectivity patterns to clarify the specific pattern of the disease in different conditions. Second, to make sure the longitudinal changes of network on the MSA patients, in the future, we will collect the data at different time point to explore the functional connectivity changes of cerebella-cerebral circuit. Third, in the future, to collect a large sample of fMRI data are essential to test the current findings. In addition, we will combine multimode MRI techniques including metabolic, perfusion and diffusion methods to provide deep understanding of the MSA mechanism.

Finally, several issues of the resting state fMRI technique need to be further discussed. First, the fMRI data is bandpass (0.01–0.08 Hz) filtered, which is applied to reduce the effects of very low frequency and high frequency physiological noise such as respiratory and aliased cardiac signals. However, the cardiac signals (usually 1.3 Hz) can not be removed completely in the long TR acquisition. Second, in the current study, all participants were instructed to close their eyes during the resting-state scans. In order to confirm that none of subjects had fallen asleep, we designed simple questionnaire after the scan, however, we can’t completely control resting state of the subjects during the scan. Sleep monitoring might be helpful in the future. Thirdly, we didn’t perform task-oriented fMRI approaches to analyze the data. The task related fMRI might provide more specific and accurate activated seed region of MSA. In the future, we will combine the task and resting state fMRI to analyze the data to provide deep understanding of MSA. At last, for resting state fMRI, seed based approach might be simple and straightforward, while, it is subjective for the selection of the seed region’s shape, size and location. Independent component analysis (ICA) is a pure data driven approach, which use statistical method to decompose data into independent components, However, there are no criteria on how many components should be identified to accurately reflect the brain networks. In this study, we initially applied the ALFF method to investigate the whole brain intrinsic activity to extract the seed region of most significant different between MSA and controls, and then we analyzed the functional connectivity based on the selective seed region. To some extent, we overcome the subjectivity of the seed region selection. Although, using different methods to confirm disruption of the cerebello-cortical circuit of the MSA is essential in the future.

## Conclusion

In conclusion, our findings provide evidence that the significant decreased spontaneous brain activity were mainly located in cerebellum in the MSA-c type patients. In addition, functional connectivity of cerebello-cortical circuit was disrupted in the MSA-c type patients, which were closely associated with the behavior performance as measured by UMSARS. These findings may be helpful for deep understanding of the mechanisms of MSA and provide a potential imaging biomarker for the diagnosis of MSA in the future.

## Author Contributions

SR and HZ wrote the manuscript. ML, WZ, and FG provided technical support. ZW and ZC reviewed the manuscript.

## Conflict of Interest Statement

The authors declare that the research was conducted in the absence of any commercial or financial relationships that could be construed as a potential conflict of interest.

## Footnotes

1 http://www.fil.ion.ucl.ac.uk/spm

2 www.restfmri.net/forum/DPARSF

3 http://restfmri.net
